# Dexmedetomidine with different concentrations added to local anesthetics in erector spinae plane block: a meta-analysis of randomized controlled trials

**DOI:** 10.3389/fmed.2024.1326566

**Published:** 2024-05-22

**Authors:** Qian Li, Yaoxin Yang, Yu Leng, Xiaowei Yin, Jin Liu, Cheng Zhou

**Affiliations:** ^1^Department of Anesthesiology, West China Hospital of Sichuan University, Chengdu, China; ^2^Laboratory of Anesthesia and Critical Care Medicine, National-Local Joint Engineering Research Centre of Translational Medicine of Anesthesiology, West China Hospital of Sichuan University, Chengdu, China

**Keywords:** dexmedetomidine, erector spinae plane block, local anesthetic, adjuvant, perioperative analgesia

## Abstract

**Background:**

Dexmedetomidine has been used as a perineural local anesthetic (LA) adjuvant to facilitate the potency of erector spinal plane block (ESPB). This quantitative review aimed to evaluate whether perineural dexmedetomidine for ESPB can improve the effects of analgesia compared to LA alone.

**Methods:**

Randomized controlled trials (RCTs) that investigated the addition of dexmedetomidine to LA compared to LA alone in ESPB were included. The pain scores, duration of sensory block, the time to first analgesia requirement, postoperative morphine consumption, rescue analgesia, and dexmedetomidine-related side effects were analyzed and combined using random-effects models.

**Results:**

A total of 823 patients from 13 RCTs were analyzed. Dexmedetomidine was used at the concentration of 0.5 μg/kg in three trials and 1 μg/kg in nine trials, and both in one trial. Both concentrations of dexmedetomidine perineurally administrated significantly reduced the rest VAS scores postoperatively at 12 h (0.5 μg/kg dexmedetomidine: MD = −0.86; 95% CI: −1.59 to −0.12; *p* = 0.02; 1 μg/kg dexmedetomidine: MD = −0.49; 95% CI: −0.83 to −0.16; *p* = 0.004), and 24 h (0.5 μg/kg dexmedetomidine: MD = −0.43; 95% CI: −0.74 to −0.13; *p* = 0.005; 1 μg/kg dexmedetomidine: MD = −0.62; 95% CI: −0.84 to −0.41; *p* < 0.00001). Both concentrations of dexmedetomidine added in LAs improved the dynamic VAS scores postoperatively at 12 h (0.5 μg/kg dexmedetomidine: MD = −0.55; 95% CI: −0.95 to −0.15; *p* = 0.007; 1 μg/kg dexmedetomidine: MD = −0.66; 95% CI: −1.05 to −0.28; *p* = 0.0006) and 24 h (0.5 μg/kg dexmedetomidine: MD = −0.52; 95% CI: −0.94 to −0.10; *p* = 0.01; 1 μg/kg dexmedetomidine: MD = −0.46; 95% CI: −0.75 to −0.16; *p* = 0.002). Furthermore, perineural dexmedetomidine prolonged the duration of the sensory block and the time to first analgesia requirement, reduced postoperative morphine consumption, and lowered the incidence of rescue analgesia and chronic pain.

**Conclusion:**

The meta-analysis showed that using perineural dexmedetomidine at either 0.5 μg/kg or 1 μg/kg doses in ESPB can effectively and safely enhance pain relief.

**Systematic review registration:**

PROSPERO (CRD42023424532: https://www.crd.york.ac.uk/PROSPERO/).

## 1 Introduction

Compared to general anesthesia and systemic analgesia, regional nerve block techniques provide important strengths, such as decreased side effects and accelerated recovery from anesthesia (1). Postoperative pain is an adverse outcome resulting in distress to the patient and an increase in the consumption of opioids for rescue analgesia. Due to concern about common and severe side effects of opioids, including sedation, nausea and vomiting, dizziness, constipation, and respiratory depression, anesthesiologists are always looking for preferable approaches to improve postoperative pain, prolong analgesia, and reduce the administration of opioids. Erector spinae plane block (ESPB), a new regional nerve block technique, is a paraspinal interfacial plane block targeting the ventral and dorsal branches of the spinal nerve under ultrasonic guidance (2, 3). This local blocking method has been proven effective for alleviating postoperative pain, decreasing postoperative opioid consumption, reducing postoperative stays in the hospital, and maintaining hemodynamic stability, with rarely corresponding complications (4–8). However, even with the administration of long-acting LAs, the analgesic effect of ESPB block with LAs alone lasts only 6 to 8 h (9).

Dexmedetomidine is a selective α_2_ agonist that has been proven safe and effective when added to LAs to prolong the analgesic effects of the regional nerve block and is accompanied by sedation, anti-anxiety, hypnosis, and inhibition of perioperative sympathetic excitation (10–12). It can prolong the duration of a single-shot block by inhibiting nerve conduction (13). Previous studies have demonstrated that adding dexmedetomidine to the brachial plexus blocks could accelerate block onset, prolong the duration of sensory block, improve the analgesic effect, and reduce morphine consumption (14). At present, the efficacy of dexmedetomidine in ESPB is not clear. Besides, its potential adverse reactions in regional nerve block, such as hypotension and bradycardia, limit its use (15). In addition, the efficacy and the side-adverse reactions of dexmedetomidine may also be dose-dependent (16).

Although previous meta-analyses have discussed the adjuvant pharmacological effects of dexmedetomidine in ESPB, they have not summarized any important benefits quantitatively in the clinic (17). Hence, our study aimed to examine the potential of dexmedetomidine at varying concentrations to enhance pain management and mitigate side effects in patients undergoing elective surgery with ESPB for perioperative analgesia.

## 2 Materials and methods

Our study followed the PRISMA (18) recommendations in preparing this manuscript. This investigation has been registered on the International Prospective Register of Systematic Reviews (PROSPERO; registration number: CRD42023424532).

### 2.1 Eligibility criteria

The inclusion criteria were as follows: (1) participants: adult patients (≥18 years) scheduled for elective surgery and received ESPB for perioperative analgesia; (2) comparison: with or without dexmedetomidine as an adjunct to local anesthetics; (3) outcomes: any treatment outcomes including postoperative visual analog scores (VAS), duration of sensory block, the time to first analgesia requirement, postoperative morphine consumption, rescue analgesia, and side effects; and (4) study design: randomized controlled trials (RCTs). We excluded trials if i.v. regional anesthetics were used (19), the control group used dexmedetomidine as well (20), or the blocks other than the erector spinae plane were performed (21, 22).

### 2.2 Search strategy

We searched for relevant studies from electronic databases, including the National Library of Medicine database, Pubmed; the Excerpta Medica database, EMBASE; the Cochrane Library database; and the Web of Science. The medical subject headings (MeSH), text word, and controlled vocabulary terms relating to dexmedetomidine were sought. Results were combined using the Boolean operator “AND” with the search terms such as erector spinal block, erector spinae, musculus erector spinae, spinal erectors, erector spinae muscles, erector, erector spinae block, ESP, ESPB, or erector spinae plane block. The result of this search was limited to randomized controlled trials and human studies published in the English language. All the trials, including adults (age > 18 years old) and published in full manuscript until May 30, 2023, were considered without any restriction of countries.

### 2.3 Risk of bias assessment

Two authors (Q.L. and Y.L.) independently evaluated the quality of the included trials using the Cochrane risk of bias tool 2.0 (RoB2) (23). This tool evaluates biases of trials, including the randomization process, deviations from the intended interventions, missing outcome data, measurement of the outcome, and selection of the reported result. Each domain is categorized as “low risk,” “some concerns,” or “high risk,” depending on the identified level of risk. The overall risk of bias is assessed as follows: “high risk of bias” if high risk is identified in any criterion, “moderate risk of bias” if there are concerns in at least one domain without high risk, and “low risk of bias” if all five criteria exhibit low risk. Each trial got a final score by consensus, and if the two authors could not reach an agreement, the third author (Y.Y.) was consulted.

### 2.4 Data extraction

The data extracted included the first author, publication year, country, surgery, sample size, types and doses of LAs, DEX concentration, block localization, nature of the primary outcome, surgical site, nerve localization technique, block characters, intraoperative and postoperative analgesic effects, and postoperative side effects. The source study text, tables, and figures were used to extract means, standard deviations (SDs), number of events, and the total number of participants. The interquartile ranges (IQR) and ranges were used for SD approximations through the formulas SD = Range/4 and SD=IQR/1.35, described by the Cochrane Handbook for Systematic Reviews (24). The data reported as 95% confidence intervals (CI) were also converted to SDs. And the means could be estimated by the data reported as medians (25). Finally, we classified the quality of evidence for each outcome using the Grades of Recommendation, Assessment, Development, and Evaluation Working Group (GRADE) system (26). The guidelines rate the power of evidence based on the risk of bias, consistency, directness, precision, and publication bias. According to these results, the strength of evidence can be divided into four levels: (1) high quality: further research is very unlikely to change our confidence in the estimate of effect; (2) moderate quality: further research is likely to have a significant impact on our confidence in the estimate of effect and may change the estimate; (3) low quality: further research is very likely to have a significant impact on our confidence in the estimate of effect and is likely to change the estimate; and (4) very low quality: we are very uncertain about the estimate.

### 2.5 Outcomes assessed

The primary outcome was the severity of rest and dynamic postoperative pain (vision analog scale, VAS; 0 = no pain, 10 = worst pain) at 12 and 24 h postoperatively. Secondary outcomes, such as other analgesic results, included time to first analgesia requirement (h), morphine consumption (mg), and rescue analgesia postoperatively. We also estimated the duration of sensory block (h), which is defined as the time from completion of LA injection to recovery of sensory block. DEX-related adverse effects, including chronic pain, hypotension, nausea and vomiting, and bradycardia, were also evaluated. Postoperative pain severity reported as a numerical rating scale (NRS) was converted to VAS scores (27).

### 2.6 Predefined source of heterogeneity

To explore the potential causes of heterogeneity in our results, we estimated the clinical characteristics of these trials and known confounders leading to variations in our outcomes. The possible sources of heterogeneity included (1) the concentration of DEX, (2) the level of ESPB and surgery, (3) the types of LAs, and (4) the dose of LAs. Different doses of DEX may generate different analgesic effects, so we planned to use subgroup analysis according to the different concentrations of DEX (0.5 μg/kg and 1 μg/kg). Meta-regression was performed for other factors.

### 2.7 Statistical analysis

We extracted the data mainly from tables. If the data was presented as a figure, we estimated them from these figures. For dichotomous outcomes, the incidence of events (n/N) was attained, and the single highest incidence was used to record the proportion of participants who experienced the events at least once. More than one intervention group that received different doses of DEX was combined into a single group based on the Cochrane Handbook (28). The odds ratio (OR) was used to pool dichotomous outcomes. Mean difference (MD) and 95% CI were used for continuous data.

### 2.8 Meta-analysis

Data entry was performed by one author (Q.L.) and checked by another (Y.L.). We used ReviewManager (Revman 5.4.1, Cochrane Library, Oxford, United Kingdom, and OpenMeta [Analyst] software version: Beta 3.13, Tufts Medical Centre) and used the R package Meta version 4.0.3 (R Foundation for Statistical Computing; Vienna, Austria) to perform statistical analyses (29). Considering potentially high methodological and clinical heterogeneity between the different trials, the random effect model was used to pool all results (30). The MD and 95% CIs were calculated for all continuous outcomes, including severity of rest and dynamic postoperative pain, duration of sensory block, the time to first analgesia, and postoperative morphine consumption. The ORs and 95% CIs were reported for the dichotomous outcomes, including the incidences of postoperative rescue analgesic, bradycardia, chronic pain, hypotension, nausea, and vomiting. The differences were considered statistically significant when the *p*-value was lower than 0.05 and 95% CI did not comprise 1 for OR and 0 for mean difference. I^2^ statistic was used to assess the heterogeneity of the pooled results (31). We explored the sources of heterogeneity of the outcome data when heterogeneity was significant (I^2^ > 50%). The publication bias was evaluated based on the asymmetry of the funnel plots according to the Egger regression test (32). We conducted sensitivity analysis by systematically excluding individual studies to assess the durability of the combined overall effects of dexmedetomidine when used as a supplement to local anesthesia in ESPB.

## 3 Results

**Figure 1:**
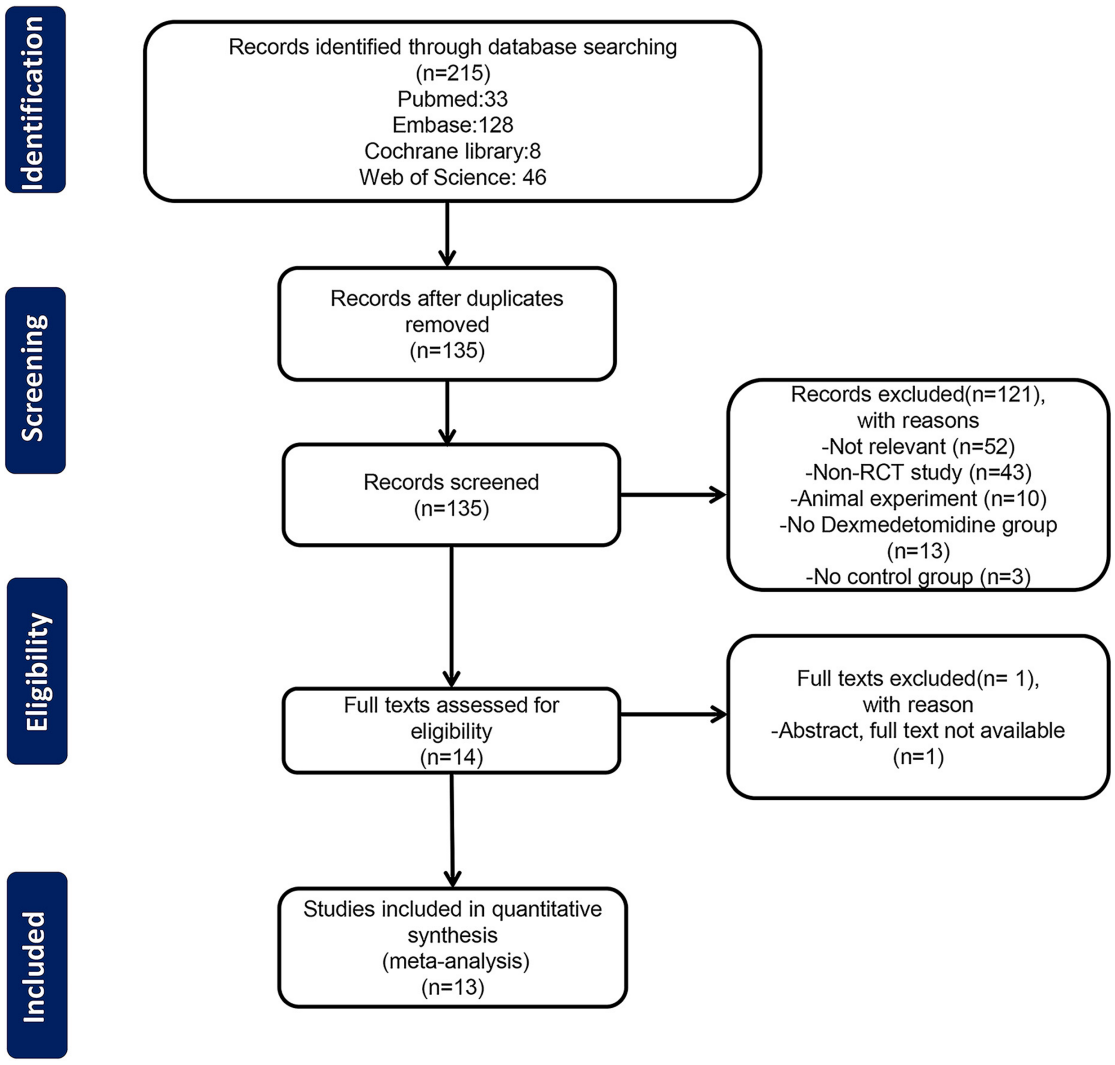
Flowchart for study selection.

We initially retrieved 215 articles after the database search. After excluding 80 duplicate studies by Endnote, the titles and abstracts of the remaining 135 articles were reviewed. Full texts of 14 potential studies were selected, but one could not be found (33). Finally, 13 full-text randomized controlled trials were included in the meta-analysis (7–9, 34–43). The detailed flowchart of the literature screening process is illustrated in Figure 1.

### 3.1 Study characteristics

Details of the 13 trials, including sample size, intervention arms, and outcomes extracted, are summarized in Table 1. In the 13 trials with 430 patients in the DEX group and 393 in the control group, ESPB was performed at different levels. In one trial, it was performed at the level of the shoulder (37); in two trials, at the level of the lumbus and abdomen (35, 36); and in 10 trials, at the level of the chest (7–9, 34, 38–43). The blocks provided surgical anesthesia in all 13 trials (7–9, 34–43). All trials used long-acting LAs, including levobupivacaine in one trial (43), bupivacaine in three trials (37, 40, 41), and ropivacaine in nine trials (7–9, 34–36, 38, 39, 42). DEX was used at a concentration of 0.5 μg/kg in three trials (9, 37, 40), 1 μg/kg in nine trials (7, 8, 34–36, 38, 41–43), and both concentrations in one trial (39). Among them, four trials (7, 34, 38, 43) also examined other adjuvants such as nalbuphine and dexamethasone, which arms were excluded from our analysis as unsuitable for the inclusion criteria. Of note, one trial used dexamethasone both in the DEX group and in the control group (39). As this trial met the initial criteria, we ultimately chose to include it in the final analysis. Considering that dexamethasone may also have an auxiliary analgesic effect, which could have caused some bias, we reanalyzed these results without this article (Supplementary Table 1).

**Table 1:** Trial characteristics and outcomes examined.

Study	Surgery	Block/use	*n*	Groups(n)	Local anesthetic concentration total volume	DEX dose (μg/kg)	Primary outcome
Ahmed 2023 (43)	MRM	Chest/surgical	90	1. Levobupivacaine+DEX (30) 2. Levobupivacaine+ dexamethasone (30) 3. Levobupivacaine (30)	0.25%-30 mL	1	Morphine consumption
Akshay 2023 (42)	VATLS	Chest/surgical	60	1. Ropivacaine+DEX (30) 2. Ropivacaine (30)	0.5%-N/D	1	N/D
Elshal 2021 (40)	Thoracic cancer surgeries	Chest/surgical	42	1. Bupivacaine+DEX (21) 2. Bupivacaine+NS (21)	0.25%-28 mL	0.5	The time first to request rescue analgesia
Gao X 2021 (39)	VATS	Chest/surgical	108	1. Ropivacaine+dexamethasone +DEX0.5 μg/kg (36) 2. Ropivacaine+dexamethasone+DEX1μg/kg (36) 3. Ropivacaine+dexamethasone (36)	0.375%-15 mL	0.5/1	The pain 12 h after surgery
Gao Z 2019 (38)	VATLS	Chest/surgical	90	1. Ropivacaine+DEX (30) 2. Ropivacaine+dexamethasone (30) 3. Ropivacaine (30)	0.5%-30 mL	1	Postoperative PCA use during the first 72 h
Hamed 2023 (37)	Shoulder arthroscopy	Shoulder/surgical	55	1. Bupivacaine+DEX (28) 2. Bupivacaine (27)	0.25%-20 mL	0.5	The total rescue morphine consumption in the first 24 postoperative hours
Hassan 2023 (41)	Breast cancer surgery	Chest/surgical	38	1. Bupivacaine+DEX (19) 2. Bupivacaine (19)	0.5%-20 mL	1	Duration of analgesia
Rao 2021 (7)	VATLS	Chest/surgical	95	1. Ropivacaine+DEX (33) 2. Ropivacaine+nalbuphine (30) 3. Ropivacaine (32)	0.5%-30 mL	1	PCA use during the first 72 h postoperatively
Sifaki 2022 (36)	Laparoscopic cholecystectomy	Lumbus and abdomen/surgical	60	1. Ropivacaine+DEX (20) 2. Ropivacaine (20) 3. NS (20)	0.375%-20 mL (each side)	1	Total postoperative morphine consumption
Wang Q 2021 (9)	Sweet procedure	Chest/surgical	60	1. Ropivacaine+DEX (30) 2. Ropivacaine+NS (30)	0.5%-28 mL	0.5	Duration of analgesia
Wang X 2021 (8)	MRM	Chest/surgical	60	1. Ropivacaine+DEX (30) 2. Ropivacaine (30)	0.33%-30 mL	1	Dosage of flurbiprofen at 48 h after surgery
Wang Y 2022 (35)	Lumbar spinal surgery	Lumbus and abdomen/surgical	120	1.Ropivacaine+DEX (44) 2. Ropivacaine (44)	0.375%-20 mL	1	VAS
Yang 2022 (34)	Thoracoscopic lobectomy with TPVB	Chest/surgical	84	1.Ropivacaine+DEX (27) 2. Ropivacaine+dexamethasone (29) 3. Ropivacaine+NS (28)	0.5%-30 mL	1	The time to the first postoperative remedial analgesia

DEX, dexmedetomidine; ml, milliliter; kg, kilogram; MRM, modified radical mastectomy; N/D, not defined; NS, normal saline; PCA, patient-controlled analgesia; TPVB, thoracic paravertebral nerve block; VAS, visual analog scale; VATS, video-assisted thoracic surgery; VATLS, video-assisted thoracoscopic lobectomy surgeries; μg, microgram.

### 3.2 Risk of bias assessment

**Figure 2:**
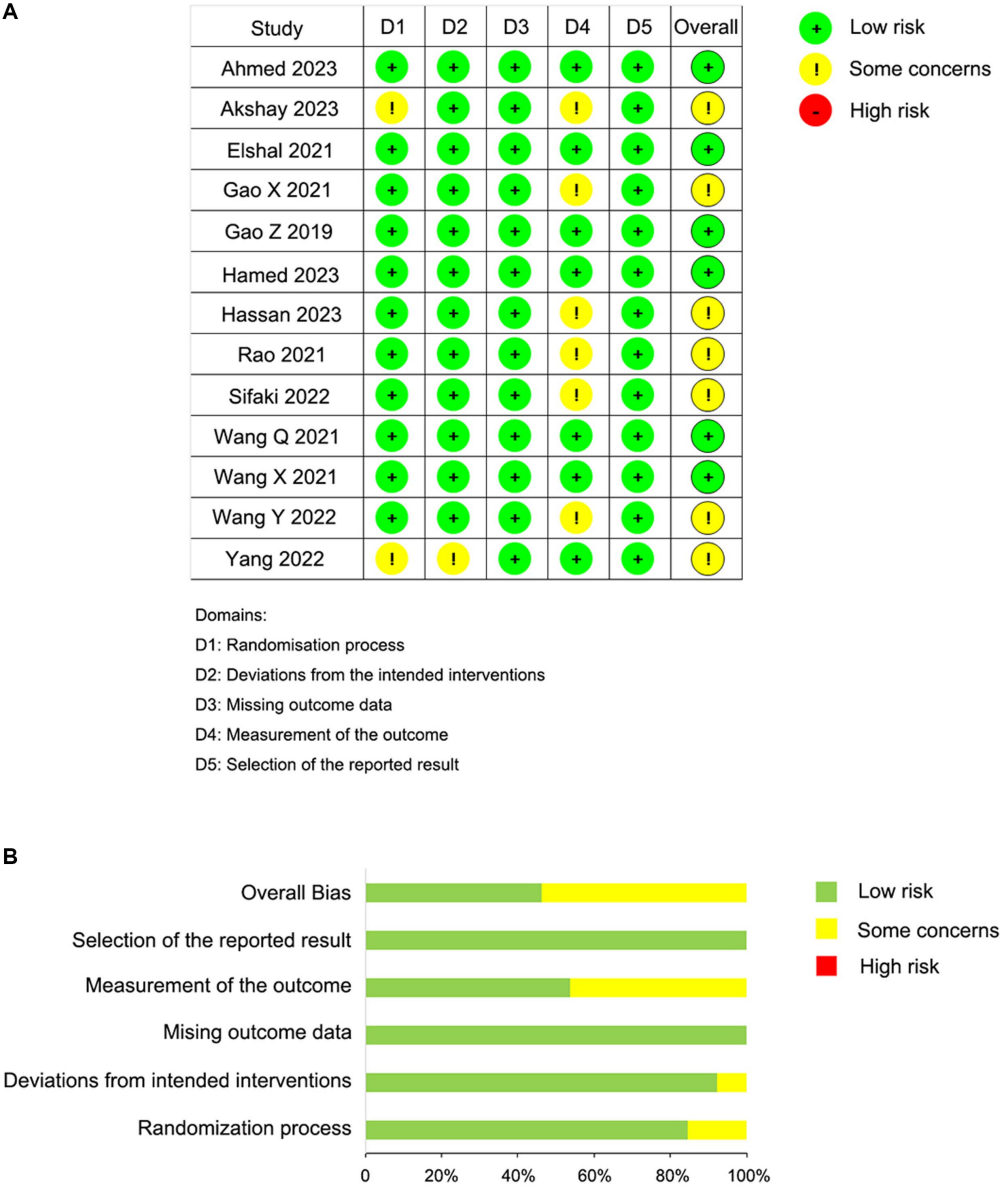
Risk of bias summary. **(A)** Risk of bias summary according to the revised Cochrane risk-of-bias tool for randomized trials (ROB 2); **(B)** Risk of bias graph according to the revised Cochrane risk-of-bias tool for randomized trials (ROB 2).

Some trials lacked enough details to fully evaluate the risk of bias; when full details allowing the exclusion of selection, performance, and detection biases were not reported, we classified these trials as having “some concerns.” The risk of bias for the 13 studies is presented in Figure 2A. Overall, the included studies were categorized as follows: six studies (46.2%) with low risk and seven studies (53.8%) with some risk (Figure 2B). Specifically, two studies exhibited some risk in the randomization process domain due to a lack of information on the randomization sequence and allocation concealment. Additionally, six studies showed some risk in the outcome measurement domain, as there was no clarity on whether outcome assessors were aware of the interventions received by participants. Other domains were deemed to have low risk.

Publication bias was evaluated by visually examining symmetry using funnel plots (Supplementary Figure 1) and Egger’s regression test for the primary outcomes. The funnel plots exhibited visual symmetry, and Egger’s regression test indicated no significant difference in the outcomes of rest and dynamic postoperative pain at 12 h/24 h postoperatively, with respective *p*-values of 0.694, 0.952, 0.805, and 0.409, which suggested the absence of publication bias.

### 3.3 Meta-analysis of primary outcome

#### 3.3.1 Rest postoperative pain severity

**Figure 3:**
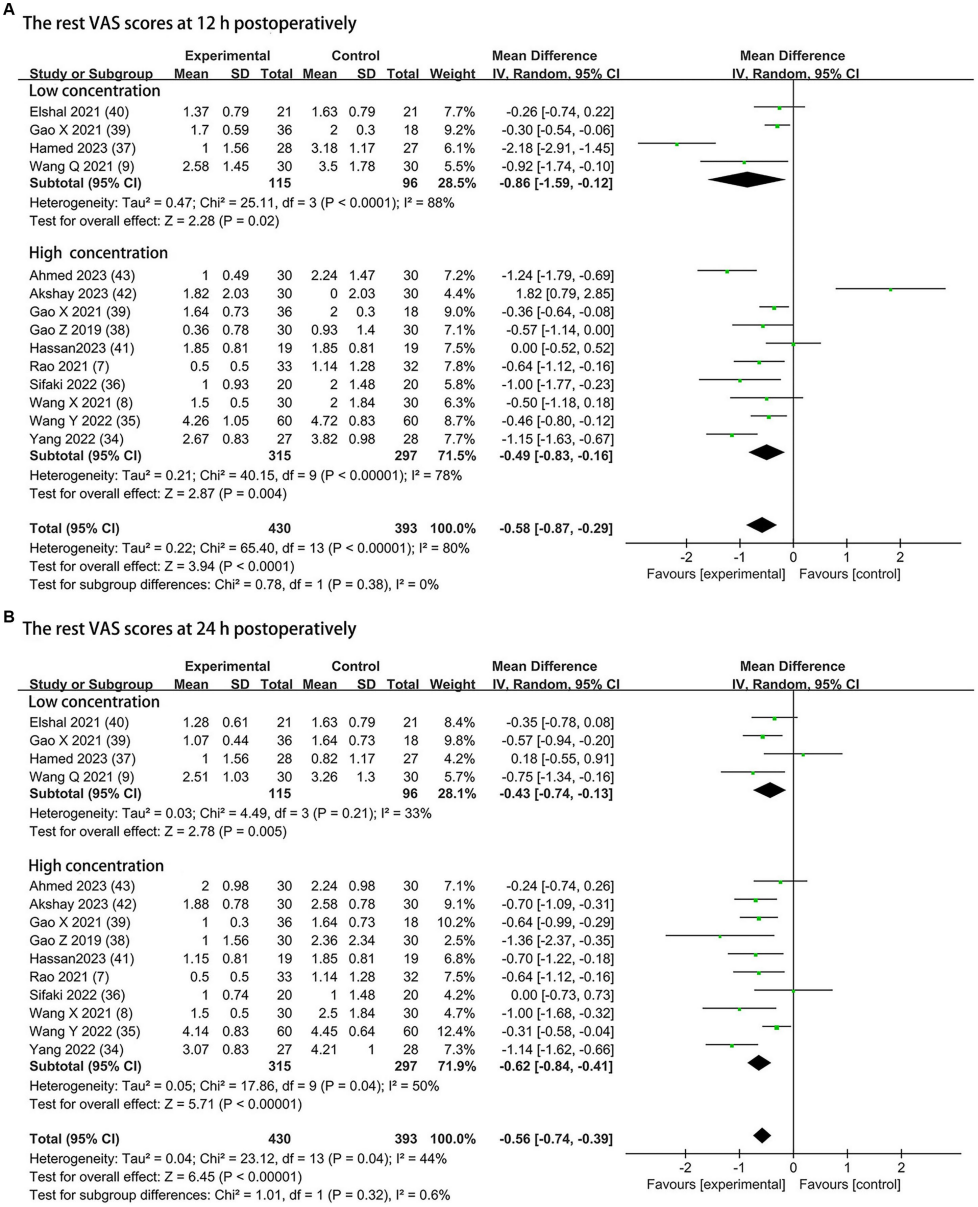
Forest plot depicting the rest pain scores: **(A)** the VAS scores at 12 h postoperatively; **(B)** the VAS scores at 24 h postoperatively. CI, Confidence interval; SD, Standard deviation; VAS, visual analog scale.

The postoperative rest pain severity (quantified by VAS) was available from all trials (430 patients in the DEX group and 393 patients in the control group), and data of the rest pain scores at 12 h and 24 h postoperatively are presented in Figure 3. Compared to the control groups, both 0.5 μg/kg or/and 1 μg/kg dexmedetomidine as an adjuvant to ESPB significantly reduced rest pain severity at 12 h postoperatively (0.5 μg/kg dexmedetomidine: MD = −0.86; 95% CI: −1.59, −0.12; *p* = 0.02; I^2^ = 88%; *p* < 0.0001; 1 μg/kg dexmedetomidine: MD = −0.49; 95% CI: −0.83, −0.16; *p* = 0.004; I^2^ = 78%; *p* < 0.00001). The overall treatment effect for both concentrations suggested that dexmedetomidine reduced rest pain severity at 12 h postoperatively (MD = −0.58; 95% CI: −0.87, −0.29; *p* < 0.0001; I^2^ = 80%; *p* < 0.00001; subgroup differences: I^2^ = 0%; *p* = 0.38; Table 2). At 24 h postoperatively, the rest postoperative pain severity of the DEX group was also lower than the control group on a DEX concentration of 0.5 μg/kg (MD = −0.43; 95% CI: −0.74, −0.13; *p* = 0.005; I^2^ = 33%; *p* = 0.21) and 1 μg/kg (MD = −0.62; 95% CI: −0.84, −0.41; *p* < 0.00001; I^2^ = 50%; *p* = 0.04). The overall treatment effect for both concentrations suggested that dexmedetomidine reduced rest pain severity at 24 h (MD = −0.56; 95% CI: −0.74, −0.39; *p* < 0.00001; I^2^ = 44%; *p* = 0.04; subgroup differences: I^2^ = 0.6%; *p* = 0.32; Table 2). Both findings were classified as having moderate levels of evidence (Table 2; Supplementary Figure 2). The overall quality assessment was downgraded by consistency limitations.

**Table 2:** Summary of results and GRADE (26) of evidence.

Time-to-event outcomes	Number of studies included	References of studies included	DEX N	DEX Mean	Control N	Control Mean	Mean Difference [95% Confidence Interval]	p-value for statistical significance	*p*-value for heterogeneity	I^2^ Test for heterogeneity	Quality of evidence (GRADE)
Rest pain scores at 12 h (cm)	13	(7–9, 34–43)	430	1.85	393	2.43	−0.58 [−0.87, −0.29]	<0.0001	<0.00001	80%	⊕ ⊕ ⊕⊝, MODERATE
Rest pain scores at 24 h (cm)	13	(7–9, 34–43)	430	1.83	393	2.49	−0.56 [−0.74, −0.39]	<0.00001	0.04	44%	⊕ ⊕ ⊕⊝, MODERATE
Dynamic pain scores at 12 h (cm)	8	(7–9, 35, 39–41, 43)	295	2.64	258	3.40	−0.63 [−0.89, −0.36]	<0.00001	0.004	64%	⊕ ⊕ ⊕⊝, MODERATE
Dynamic pain scores at 24 h (cm)	8	(7–9, 35, 39–41, 43)	295	2.88	258	3.57	−0.48 [−0.70, −0.25]	<0.0001	0.06	46%	⊕ ⊕ ⊕⊝, MODERATE
Analgesic outcomes	Number of studies included	References of studies included	DEX N	DEX (Mean or n/N)	Control N	Control (Mean or n/N)	Mean Difference [95% Confidence Interval]	*p*-value for statistical significance	*p*-value for heterogeneity	I^2^ test for heterogeneity	Quality of evidence (GRADE)
Duration of sensory block (h)	3	(7, 38, 43)	93	15.98	92	9.87	5.69 [2.19, 9.19]	0.001	0.002	84%	⊕ ⊕ ⊕⊝, MODERATE
Time to first analgesia requirement (h)	9	(7, 9, 34, 37–41, 43)	268	14.44	241	8.55	5.96 [4.08, 7.84]	<0.00001	<0.00001	92%	⊕ ⊕ ⊕⊝, MODERATE
Postoperative morphine consumption (mg)	5	(36, 37, 40, 41, 43)	118	3.63	117	5.41	−1.79 [−2.45, −1.13]	<0.00001	0.35	10%	⊕ ⊕ ⊕⊕, HIGH
Rescue analgesia	6	(7, 37–40, 43)	214	51/214	176	82/176	0.30 [0.17, 0.53]	<0.0001	0.37	8%	⊕ ⊕ ⊝⊝, LOW
DEX-related adverse effect	Number of studies included	References of studies included	DEX N	DEX (Mean or n/N)	Control N	Control (Mean or n/N)	Mean Difference [95% Confidence Interval]	*p*-value for statistical significance	*p*-value for heterogeneity	I^2^ Test for heterogeneity	Quality of evidence (GRADE)
Chronic pain	2	(7, 39)	105	12/105	68	22/68	0.24 [0.10, 0.56]	0.001	0.34	0%	⊕⊝⊝⊝, VERY LOW
Hypotension	6	(8, 9, 34, 37, 39, 42)	217	19/217	181	12/181	1.22 [0.57, 2.60]	0.62	0.89	0%	⊕ ⊕ ⊕⊝, MODERATE
Nausea and Vomiting	12	(7–9, 34, 36–43, 45)	370	70/370	333	77/333	0.68 [0.46, 1.01]	0.06	0.78	0%	⊕ ⊕ ⊕⊕, HIGH
Bradycardia	6	(8, 9, 34, 37, 39, 42)	217	24/217	181	21/181	0.81 [0.42, 1.58]	0.54	0.43	0%	⊕ ⊕ ⊕⊝, MODERATE

cm, centimeter; DEX, dexmedetomidine; h, hour; mg, milligram.

#### 3.3.2 Dynamic postoperative pain severity

**Figure 4:**
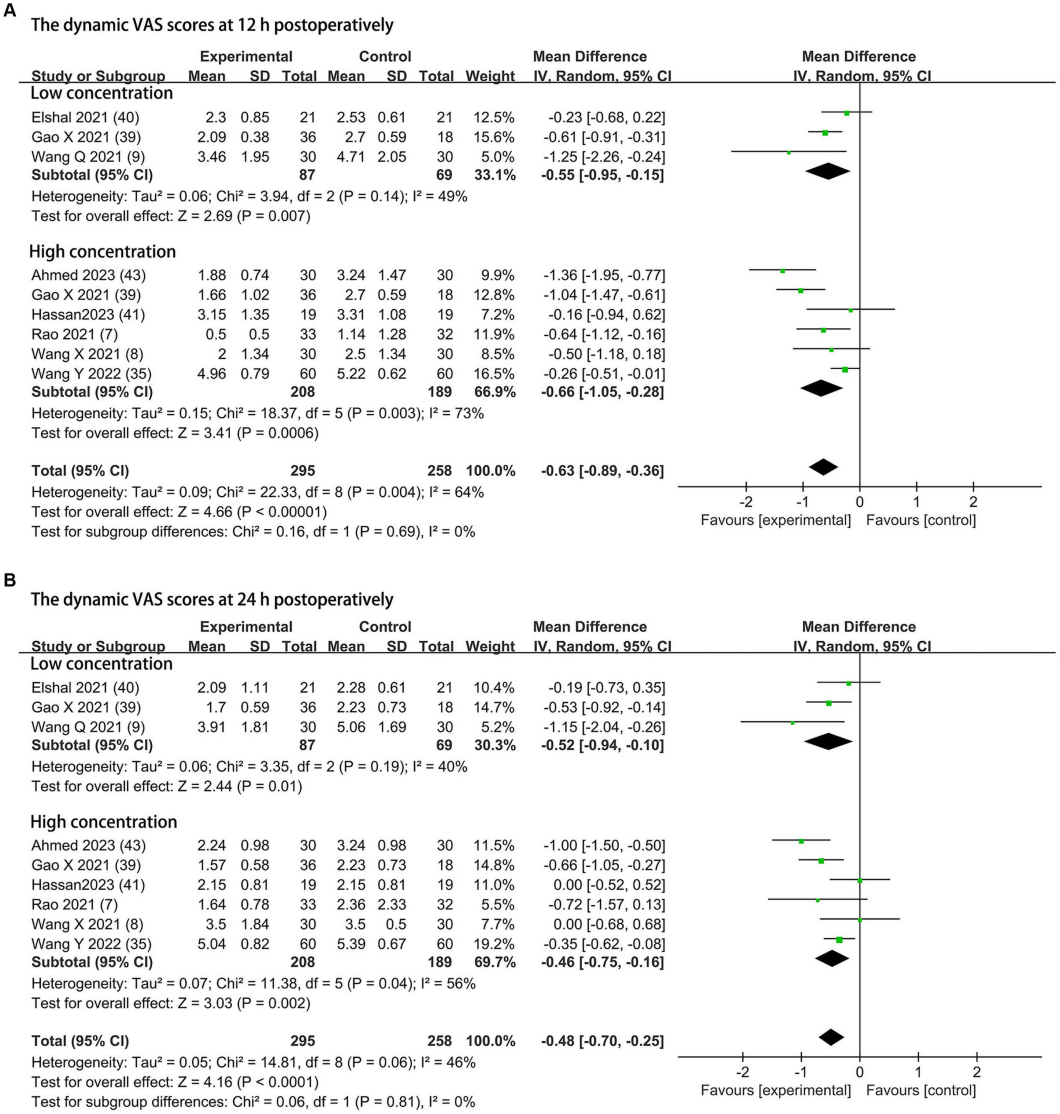
Forest plot depicting the dynamic pain scores: **(A)** the VAS scores at 12 h postoperatively; **(B)** the VAS scores at 24 h postoperatively. CI, Confidence interval; SD, Standard deviation; VAS, visual analog scale.

Dynamic pain scores at 12 h and 24 h postoperatively were available from eight trials (295 patients in the DEX group and 258 patients in the control group) and are shown in Figure 4 (7–9, 35, 39–41, 43). Compared to the control groups, dexmedetomidine as an adjuvant to ESPB reduced the dynamic postoperative pain severity at 12 h at concentrations of 0.5 μg/kg (MD = −0.55; 95% CI: −0.95, −0.15; *p* = 0.007; I^2^ = 49%; *p* = 0.14) and 1 μg/kg (MD = −0.66; 95% CI: −1.05, −0.28; *p* = 0.0006; I^2^ = 73%; *p* = 0.003). The overall treatment effect of both concentrations suggested that dexmedetomidine reduced dynamic pain severity at 12 h (MD = −0.63; 95% CI: −0.89, −0.36; *p* < 0.00001; I^2^ = 64%; *p* = 0.004; subgroup differences: I^2^ = 0%; *p* = 0.69; Table 2). At 24 h, the dynamic postoperative pain severity was lower in DEX group than in the control group on concentrations of 0.5 μg/kg (MD = −0.52; 95% CI: −0.94, −0.10; *p* = 0.01; I^2^ = 40%; *p* = 0.19) and 1 μg/kg (MD = −0.46; 95% CI: −0.75, −0.16; *p* = 0.002; I^2^ = 56%; *p* = 0.04). The overall treatment effect for both concentrations suggested that dexmedetomidine reduced dynamic pain severity at 24 h (MD = −0.48; 95% CI: −0.70, −0.25; *p* < 0.0001; I^2^ = 46%; *p* = 0.06; subgroup differences: I^2^ = 0%; *p* = 0.81; Table 2). Both findings were classified as moderate levels of evidence (Table 2; Supplementary Figure 2). The overall quality assessment was downgraded by consistency limitations.

### 3.4 Meta-analysis of secondary outcomes

#### 3.4.1 Duration of sensory block

**Figure 5:**
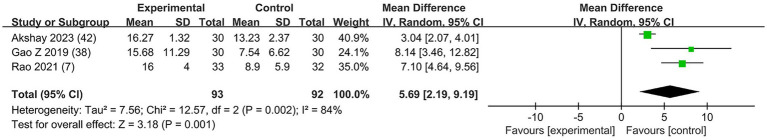
Forest plot depicting the duration of sensory block. CI, Confidence interval; SD, Standard deviation.

The effects of dexmedetomidine added to LAs in ESPB on the duration of sensory block were evaluated in three trials (7, 38, 42). All trials used dexmedetomidine with a concentration of 1 μg/kg. Dexmedetomidine as an adjuvant in ESPB prolonged the mean sensory block duration (MD = 5.69; 95% CI: 2.19, 9.19; *p* = 0.001; I^2^ = 84%; *p* = 0.002; Figure 5). The level of evidence for this finding was rated as moderate (Table 2; Supplementary Figure 2). The overall quality assessment was downgraded by consistency and sparse data limitations, but it was also upgraded by the large treatment effect.

#### 3.4.2 The time to first analgesia

**Figure 6:**
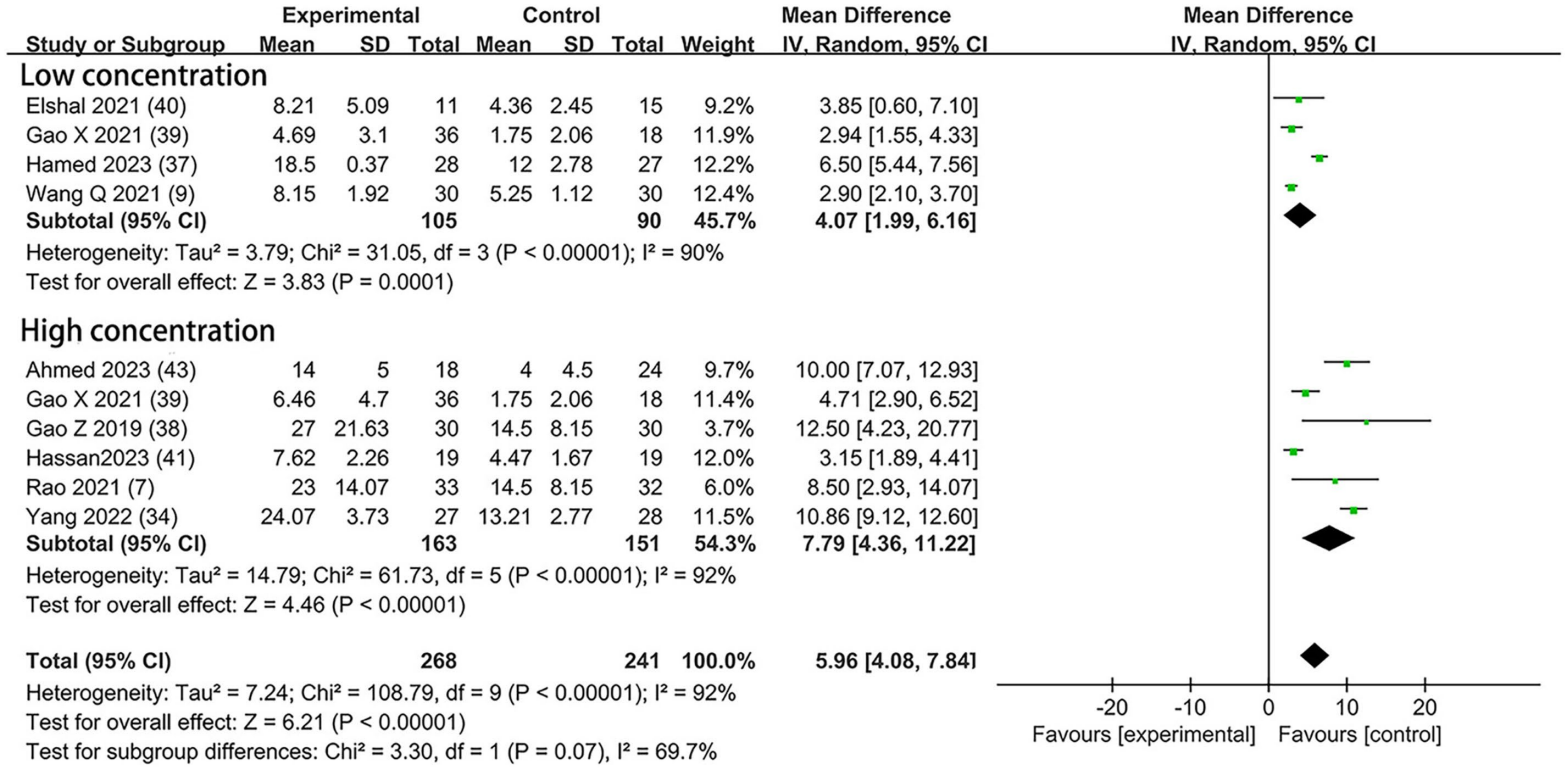
Forest plot depicting the time to first analgesia. CI, Confidence interval; SD, Standard deviation.

Nine studies included the outcome of the time to first analgesia, including three trials (9, 37, 40) with 0.5 μg/kg DEX concentration, five trials (7, 34, 38, 41, 43) with 1 μg/kg DEX concentration, and one trial (39) with both low and high concentrations. In the subgroup that received low concentration of dexmedetomidine, the time to first analgesia requirement was prolonged by 4.07 h (95% CI:1.99, 6.16; *p* = 0.0001; I^2^ = 90%; *p* < 0.00001); in the high concentration of dexmedetomidine subgroup, it was prolonged by 7.79 h (95% CI: 4.36, 11.22; *p* < 0.00001; I^2^ = 92%; *p* < 0.00001; Figure 6). The overall treatment effect for both concentrations suggested that dexmedetomidine prolonged the time to first analgesia (MD = 5.96; 95% CI: 4.08, 7.84; *p* < 0.00001; I^2^ = 92%; *p* < 0.00001; subgroup differences: I^2^ = 69.7%; *p* = 0.07; Table 2). The level of evidence for this finding was rated as moderate (Table 2; Supplementary Figure 2). The overall quality assessment was downgraded by consistency limitations and publication bias, but it was also upgraded by the dose–response effect.

#### 3.4.3 Postoperative morphine consumption

**Figure 7:**
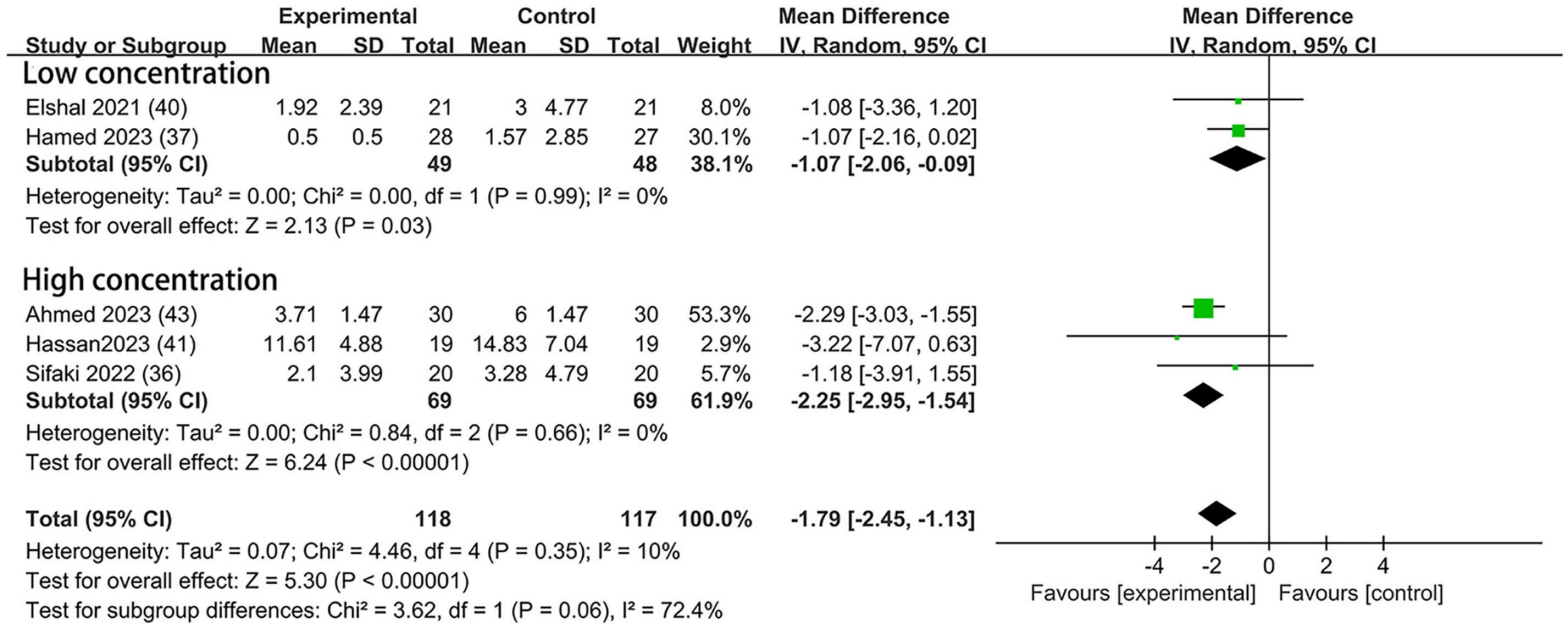
Forest plot depicting the postoperative morphine consumption. CI, Confidence interval; SD, Standard deviation.

Morphine was used for postoperative analgesia in five trials—two (37, 40) with low concentration and three (36, 41, 43) with high. Compared to the control groups, the consumption of morphine in the DEX groups with low concentration was reduced by 1.07 mg (95% CI: −2.06, −0.09; *p* = 0.03; I^2^ = 0%; *p* = 0.99); in the high concentration subgroup, the consumption of morphine was reduced by 2.25 mg (95% CI: −2.95, −1.54, *p* < 0.00001; I^2^ = 0%; *p* = 0.66; Figure 7). The overall treatment effect for both concentrations suggested that dexmedetomidine reduced the postoperative morphine consumption (MD = −1.79; 95% CI: −2.45, −1.13; *p* < 0.00001; I^2^ = 10%; *p* = 0.35; subgroup differences: I^2^ = 72.4%; *p* = 0.06; Table 2). The finding was classified as high levels of evidence (Table 2; Supplementary Figure 2). The overall quality assessment was downgraded by sparse data limitations, but it was also upgraded by the dose–response effect.

#### 3.4.4 Incidence of postoperative rescue analgesic

**Figure 8:**
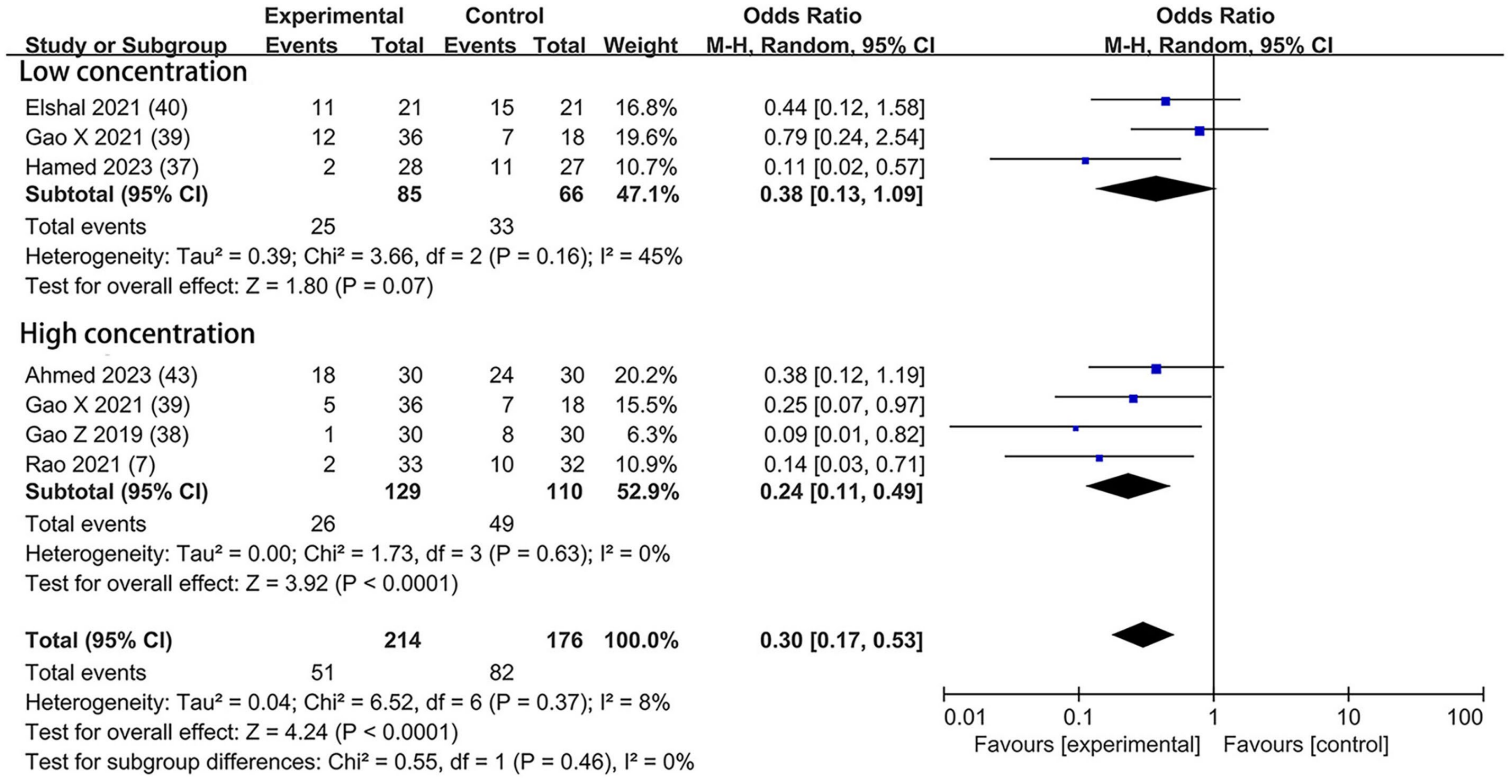
Forest plot depicting the incidence of postoperative rescue analgesic. CI, Confidence interval.

The event of postoperative rescue analgesic was explicitly assessed in six trials—two with the dexmedetomidine of 0.5 μg/kg (37, 40), three with the dexmedetomidine of 1 μg/kg (7, 38, 43), and one with both concentrations (39). A lower incidence of postoperative rescue analgesic was noticed among patients who received the higher concentration of dexmedetomidine (OR = 0.24; 95% CI: 0.11, 0.49; *p* < 0.0001; I^2^ = 0%; *p* = 0.63) but not at the lower concentration (OR = 0.38; 95% CI: 0.13, 1.09; *p* = 0.07; I^2^ = 45%; *p* = 0.16; Figure 8). The overall treatment effect for both concentrations suggested that dexmedetomidine reduced the incidence of postoperative rescue analgesic (MD = 0.30; 95% CI: 0.17, 0.53; *p* < 0.0001; I^2^ = 8%; *p* = 0.37; subgroup differences: I^2^ = 0%; *p* = 0.46; Table 2). The level of evidence for this finding was rated as low (Table 2; Supplementary Figure 2). The overall quality assessment was downgraded by sparse data limitations and publication bias limitations.

#### 3.4.5 Dexmedetomidine-related adverse effects

**Figure 9:**
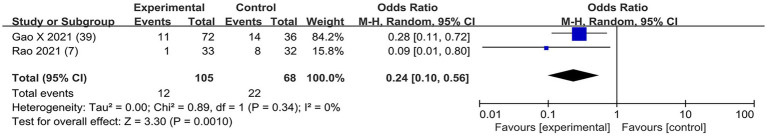
Forest plot depicting the incidence of postoperative chronic pain. CI, Confidence interval.

**Figure 10:**
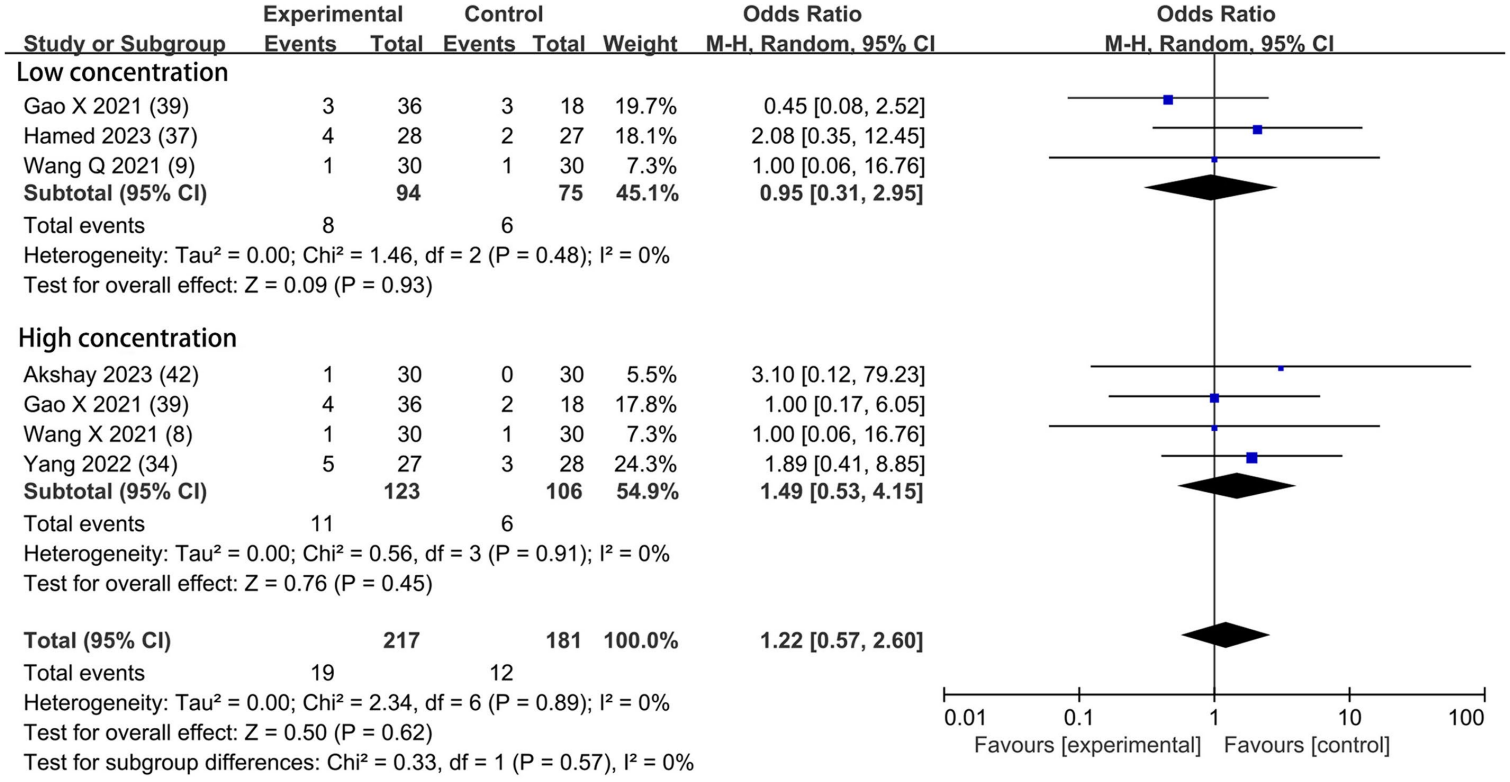
Forest plot depicting the incidence of postoperative hypotension. CI, Confidence interval.

**Figure 11:**
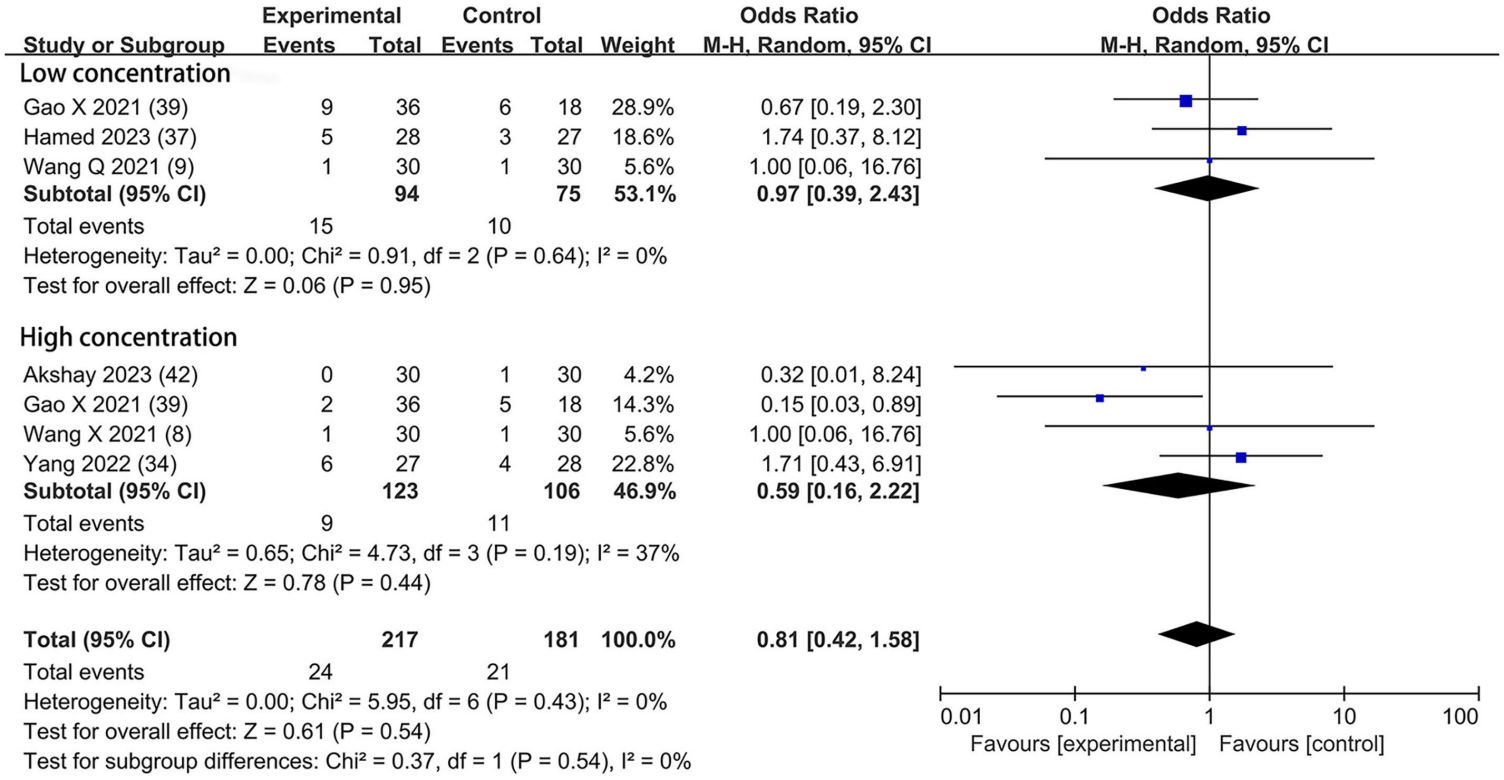
Forest plot depicting the incidence of postoperative bradycardia. CI, Confidence interval.

**Figure 12:**
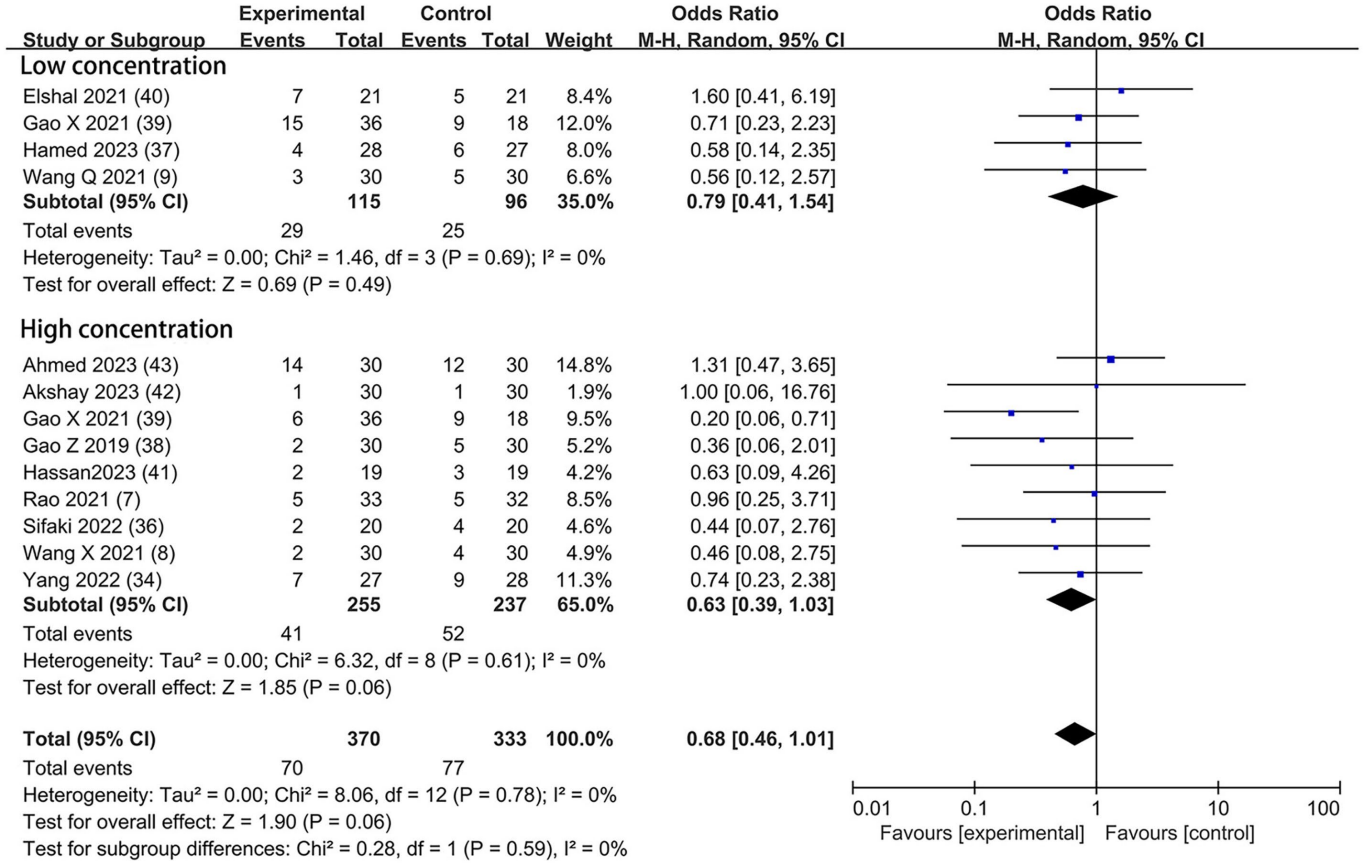
Forest plot depicting the incidence of postoperative nausea and vomiting. CI, Confidence interval.

Based on the diversity in the definitions of dexmedetomidine-related adverse effects in the reviewed trials, we reported the outcomes as “standardized units.” Chronic pain was assessed in two trials (7, 39), defined as pain for over 3 months. The incidence of chronic pain was lower in DEX groups compared with the control (OR = 0.24; 95% CI: 0.10, 0.56; *p* = 0.0010; I^2^ = 0%; *p* = 0.34; Figure 9). The level of evidence for this finding was rated as very low (Table 2; Supplementary Figure 2). The overall quality assessment was downgraded by sparse data limitations and publication bias limitations. Hypotension (Figure 10) and the incidence of bradycardia (Figure 11) were evaluated in six trials (8, 9, 34, 37, 39, 42). These results suggested that there was no significance between the DEX and control groups. Both findings were classified as moderate levels of evidence (Table 2; Supplementary Figure 2). The overall quality assessments were both downgraded by sparse data limitations. The incidence of nausea and vomiting was evaluated in all the trials except one (35) (Figure 12). There was no statistically significant difference in the outcome incidence compared with control groups. This finding’s quality was rated high (Table 2; Supplementary Figure 2). The overall quality assessment was not downgraded.

### 3.5 Sensitivity analysis

Given the significant heterogeneity and risk of bias, sensitivity analyses were performed to assess the stability of the combined results. Excluding the shoulder joint study (37) led to changes in the rest pain scores at 12 h (MD = −0.48; 95% CI: −0.74 to −0.23; *p* = 0.0002; I^2^ = 73%; *p* < 0.0001) and at 24 h (MD = −0.59; 95% CI: −0.76 to −0.43; *p* < 0.00001; I^2^ = 38%; *p* = 0.08), reducing heterogeneity by 7 and 6%, respectively. After excluding the study on lumbar spine surgery (35), rest pain scores at 12 h showed an MD of −0.59 (95% CI: −0.91 to −0.27; *p* = 0.0003; I^2^ = 82%; *p* < 0.00001) and at 24 h (MD = −0.60; 95% CI: −0.78 to −0.42; p < 0.00001; I^2^ = 38%; *p* = 0.08); dynamic postoperative pain at 12 h (MD = −0.70; 95% CI: −0.97 to −0.42; *p* < 0.00001; I^2^ = 54%; *p* = 0.03) and at 24 h (MD = −0.51; 95% CI: −0.78 to −0.23; *p* = 0.0003; I^2^ = 49%; *p* = 0.05), with heterogeneity changes of −2, 6, 10%, and − 3%. Removing the abdominal surgery study (36) impacted the 12-h rest pain scores (MD = −0.56; 95% CI: −0.86 to −0.26; *p* = 0.0003; I^2^ = 81%; *p* < 0.00001) and the 24-h scores (MD = −0.59; 95% CI: −0.76 to −0.42; *p* < 0.00001; I^2^ = 43%; *p* = 0.05), with a minimal heterogeneity reduction of −1 and 1%. Despite these exclusions, high heterogeneity persisted, potentially due to varying disease severity and surgical procedures among the patients.

## 4 Discussion

In the meta-analysis, we ultimately included 13 eligible articles (7–9, 34–43) to assess the impact of adding dexmedetomidine into ESPB. Our review demonstrated that using dexmedetomidine perineurally as an ESPB adjuvant was connected with important effects regardless of the concentration used. In particular, it reduced postoperative pain severity, extended duration of sensory block, decreased time to first request for pain relief, reduced consumption of morphine, lowered incidence of postoperative rescue analgesic, and reduced occurrence of chronic pain without affecting the risk of nausea and vomiting, bradycardia, and hypotension.

Regional anesthesia is an important part of a comprehensive pain relief plan, which includes neuraxial anesthesia and peripheral nerve block techniques. With the advancement of ultrasound-guided regional anesthesia, the fascial plane block has been utilized. This technique involves injecting a local anesthetic into musculofascial planes that include nerves rather than directly around specific nerves, and the injection is done at a distance from important structures such as major vessels, the spinal cord, or the pleura (45). As it is easy to perform and has an appealing safety profile, the ultrasound-guided fascial plane block is used increasingly in numbers and types, such as transverse abdominal plane block and ESPB. ESPB was first reported in thoracic surgery in 2016 to treat pain (2). As reported in the articles published, this technique has shown promising results in treating pain and was relatively easy to perform with a low risk of complications (46, 47). However, the duration of pain relief is still insufficient (48).

As an adjunct for anesthesia, dexmedetomidine can be administered via different routes, including wound infiltration, perineural, neuraxial, and general, to prolong the duration of intravenous regional anesthesia, peripheral nerve blocks, and spinal analgesia (49). There are many other adjuvants with similar effects to dexmedetomidine, such as opioids, dexamethasone, and ketamine. However, some trials have reported that side effects, such as nausea, vomiting, and itching, can be caused by opioids (50). Dawson and colleagues discovered that using dexamethasone as an additive might excessively prolong the pain relief period and lead to slower postoperative recovery of movement (51). Additionally, ketamine has been found to cause various adverse reactions, including nausea, drowsiness, and hallucinations (52). Therefore, dexmedetomidine offers certain advantages over other additives.

In this meta-analysis, we found that dexmedetomidine significantly reduced both the rest and dynamic postoperative VAS scores. Interestingly, we did not observe a significant difference in pain relief between the 1.0 μg/kg and 0.5 μg/kg doses of dexmedetomidine. In the present meta-analysis, although the mean difference in VAS score was relatively small at approximately 0.5 cm, our findings suggested that adding dexmedetomidine to postoperative pain management protocols might still be beneficial. Since the VAS score was just one way we measured pain relief, our other findings—such as the marked decrease in post-surgery morphine use, lower need for additional pain relief after surgery, and longer intervals before needing additional pain relief after adding dexmedetomidine—all demonstrated the clinical importance of dexmedetomidine in easing postoperative pain. In 2015, a meta-analysis focusing on dexmedetomidine administration in adults also reported a reduction in postoperative pain intensity (53). Additionally, dexmedetomidine was found to be effective as a standalone analgesic for postoperative pain relief (54).

Our research revealed that a 1 μg/kg dose of dexmedetomidine significantly prolonged the duration of sensory block. Moreover, dexmedetomidine extended the time to the first request for analgesia and decreased the use of postoperative morphine at both 0.5 μg/kg and 1 μg/kg doses. Notably, the mean difference in the 1 μg/kg group was larger than in the 0.5 μg/kg group; however, the differences between the two groups did not reach statistical significance. Dexmedetomidine achieves its sedative and analgesic effects by interacting with central presynaptic and postsynaptic α-2 receptors, and these effects are dependent on concentrations within the range of 0.2 to 0.3 ng/mL (55). Some trials have suggested that a higher dose of dexmedetomidine may improve analgesic effects in arthroscopic surgeries, including longer duration of sensory block, longer time to the first request for analgesia, and smaller consumption of postoperative morphine (56, 57).

Furthermore, it has been reported that adding dexmedetomidine to a local anesthetic solution blocking inferior alveolar nerve in the study of testing pulp latency and lower lip numbness can decrease the need for rescue analgesia, which aligns with our findings (16). Dexmedetomidine provides a stronger pain-relieving effect compared to medications such as acetaminophen and clonidine, making it an attractive option for patients dealing with chronic pain (58, 59). Our research found that dexmedetomidine as an adjunct to ESPB could reduce the incidence of chronic pain. Previous studies have suggested that using perineural dexmedetomidine may lower the risk of nausea and vomiting (53), but it could also increase the chances of hypotension and bradycardia (14, 60). However, other research has indicated that perineural dexmedetomidine does not affect the occurrence of nausea, vomiting, low blood pressure, or slow heart rate (16, 44). In our study, the use of dexmedetomidine as an additional treatment in ESPB did not impact the occurrence of these events. Dexmedetomidine elicits a biphasic hemodynamic response, with high doses potentially causing tachycardia and elevated blood pressure, while low doses may lead to bradycardia and hypotension (61). Therefore, the differences in findings may be due to variations in the nerve block site and the dosage of dexmedetomidine.

Our research has several limitations. First, some outcomes still have high heterogeneity despite us stratifying the studies according to the concentration of dexmedetomidine. Different surgical, anesthetic, and analgesic settings and different physical conditions of patients, are likely to result in clinical heterogeneity. We lack enough data to undertake more stratified studies. Second, the sample of trials we included was small, which increased the chances of bias. Third, our study had only two types of DEX concentration; more concentrations need to be included. Fourth, most of the trials were from China and Egypt, which may be a source of publication bias. Fifth, we did not search for articles from clinical trial registries; we just included trials in English. Sixth, one trial (39) utilized dexamethasone in all study arms. We ultimately decided to include this study in our analysis. This decision was based on the fact that the results obtained with and without the exclusion of this study did not differ significantly, aligning with our initial inclusion and exclusion criteria. This decision might inevitably bring potential bias.

Our review has several advantages. The articles’ literature retrieval, screening, and inclusion were exhaustive. Only randomized trials were included. Though statistical heterogeneity was explored, our outcome results maintained their robustness, emphasizing our findings’ validity.

## 5 Conclusion

In conclusion, our review provides evidence that using perineural dexmedetomidine at a dose concentration of 0.5 μg/kg or 1 μg/kg in ESPB reduces postoperative pain severity, extends the duration of sensory block, decreases the time to first request for pain relief, reduces the consumption of morphine, lowers incidence of postoperative rescue analgesic, reduces the occurrence of chronic pain, and does not affect the risk of nausea and vomiting, bradycardia, and hypotension. However, it is important to interpret this result cautiously due to the significant variability between studies. Further well-designed studies with a larger sample size are necessary to confirm the effectiveness and safety of dexmedetomidine.

## Data availability statement

The original contributions presented in the study are included in the article/Supplementary material, further inquiries can be directed to the corresponding authors.

## Author contributions

QL: Methodology, Writing – original draft. YY: Funding acquisition, Project administration, Writing – review & editing. YL: Methodology, Writing – original draft. XY: Methodology, Writing – original draft. JL: Writing – review & editing. CZ: Writing – review & editing.
