# Supplementary material for: Dexmedetomidine with different concentrations added to local anesthetics in erector spinae plane block: a meta-analysis of randomized controlled trials
Source: Front Med (Lausanne). 2024 May 22;11:1326566. doi: 10.3389/fmed.2024.1326566 (PMC11150627; doi:10.3389/fmed.2024.1326566)
Supplement: Supplementary file 1 [file Data_Sheet_1.docx]

Supplementary Material

Dexmedetomidine with different concentrations added to local anesthetics in Erector Spinae Plane Block: A Meta-Analysis of Randomized Controlled Trials

**Qian Li1,2 †, Yaoxin Yang1,2 †, Yu Leng1,2, Xiaowei Yin1, Jin Liu2* and Cheng Zhou2***

*** Correspondence:** Dr. Cheng Zhou: [zhouc@163.com](mailto:zhouc@163.com)；Dr. Jin Liu: [scujinliu@foxmail.com](mailto:scujinliu@foxmail.com)

# Supplementary Figures and Tables

## Supplementary Figures


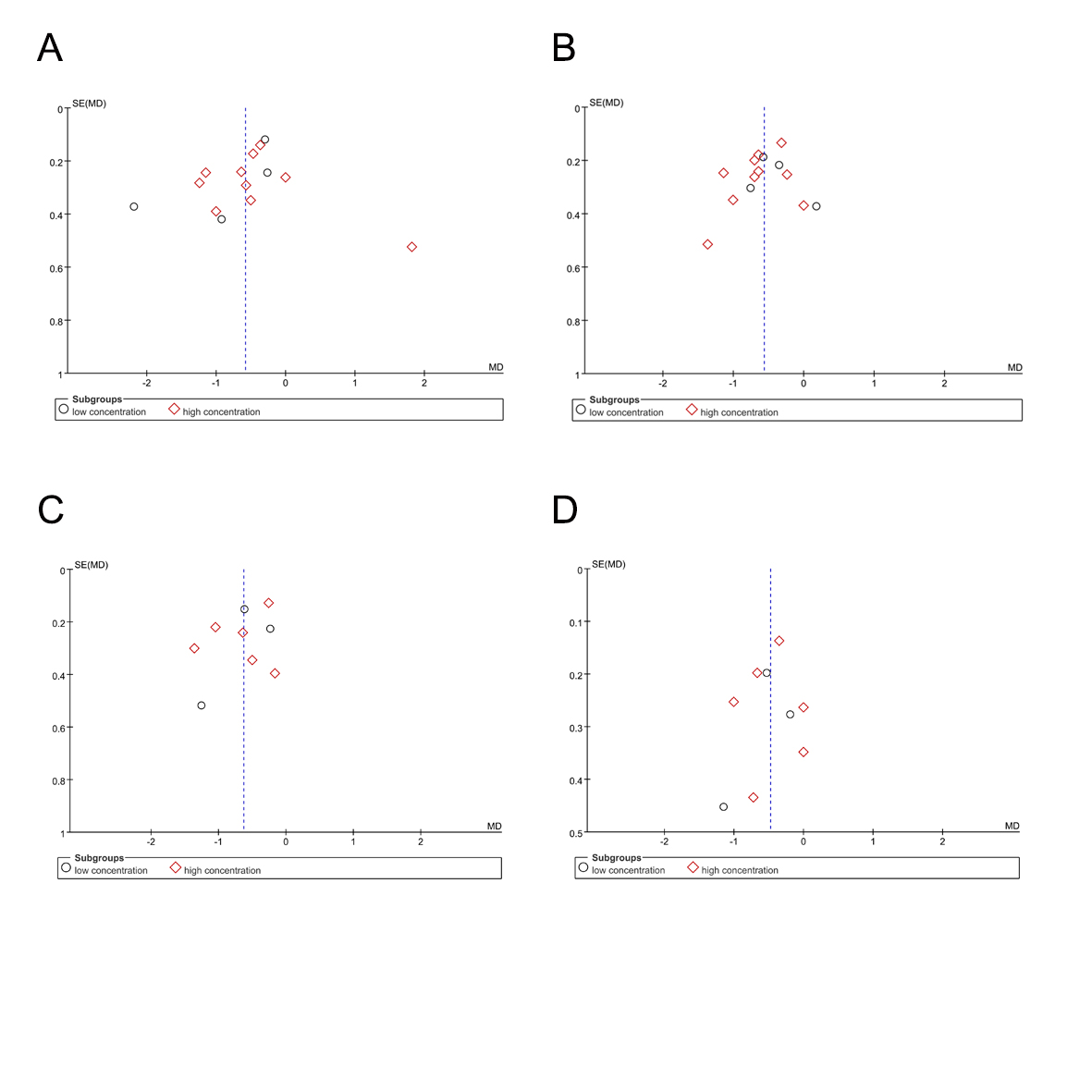


**Supplementary Figure 1.** Funnel plots of primary outcomes. (A) Funnel plot depicting the rest pain scores at 12 hours postoperatively; (B) Funnel plot depicting the rest pain scores at 24 hours postoperatively; (C) Funnel plot depicting the dynamic pain scores at 12 hours postoperatively; (D) Funnel plot depicting the dynamic pain scores at 24 hours postoperatively. SE, Standard Error; MD, Mean Difference


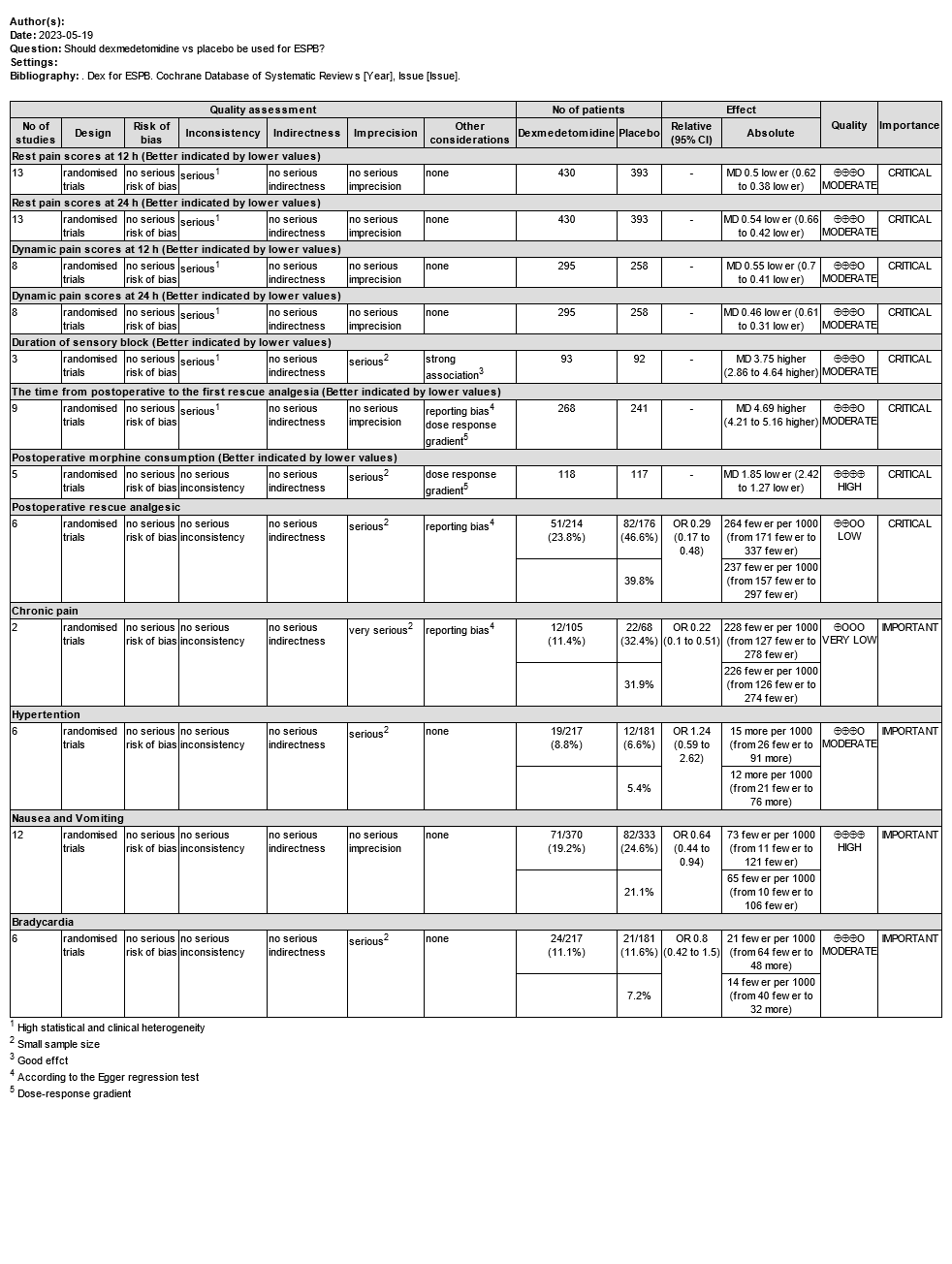


**Supplementary Figure 2.** GRADE of evidence.

## Supplementary Tables

**Supplementary Table 1**: Summary of results and GRADE of evidence.

| Time-to-event outcomes | Number of studies  included | References of studies included | DEX N | DEX Mean | Control  N | Control  Mean | Mean Difference  [95% Confidence Interval] | P-Value for statistical significance | P-Value for  heterogeneity | I^2^ Test for  heterogeneity | Quality of evidence  (GRADE) |
| --- | --- | --- | --- | --- | --- | --- | --- | --- | --- | --- | --- |
| Rest pain scores at 12h (cm) | 12 | 7,8,9,34,35,36,37,38,40,41,42,43 | 358 | 1.88 | 357 | 2.47 | -0.63[-1.01, -0.25] | 0.001 | <0.00001 | 81% | ⊕⊕⊕⊝, MODERATE |
| Rest pain scores at 24h (cm) | 12 | 7,8,9,34,35,36,37,38,40,41,42,43 | 358 | 1.99 | 357 | 2.58 | -0.56 [-0.77, -0.35] | <0.00001 | 0.02 | 52% | ⊕⊕⊕⊝, MODERATE |
| Dynamic pain scores at 12h (cm) | 7 | 7,8,9,35,40,41,43 | 223 | 2.88 | 222 | 3.51 | -0.56 [-0.89, -0.24] | 0.0007 | 0.01 | 62% | ⊕⊕⊕⊝, MODERATE |
| Dynamic pain scores at 24h (cm) | 7 | 7,8,9,35,40,41,43 | 223 | 3.28 | 222 | 3.79 | -0.44 [-0.74, -0.13] | 0.005 | 0.04 | 55% | ⊕⊕⊕⊝, MODERATE |
| Analgesic outcomes | Number of studies  included | References of studies included | DEX  N | DEX (Mean or n/N) | Control  N | Control  (Mean or n/N) | Mean Difference  [95% Confidence Interval] | P-Value for statistical significance | P-Value for  heterogeneity | I^2^ Test for  heterogeneity | Quality of evidence  (GRADE) |
| Duration of sensory block (h) | 3 | 7,38,42 | 93 | 15.98 | 92 | 9.87 | 5.69 [2.19, 9.19] | 0.001 | 0.002 | 84% | ⊕⊕⊕⊝, MODERATE |
| Time to first analgesia requirement (h) | 8 | 7,9,34,37,38,40,41,43 | 196 | 17.70 | 205 | 9.74 | 6.69 [4.30, 9.08] | <0.00001 | <0.00001 | 93% | ⊕⊕⊕⊝, MODERATE |
| Postoperative morphine consumption (mg) | 5 | 36,37,40,41,43 | 118 | 3.63 | 117 | 5.41 | -1.79 [-2.45, -1.13] | <0.00001 | 0.35 | 10% | ⊕⊕⊕⊕, HIGH |
| Rescue analgesia | 5 | 7,37,38,40,43 | 142 | 34/142 | 140 | 68/140 | 0.24 [0.13, 0.46] | <0.0001 | 0.49 | 0% | ⊕⊕⊝⊝, LOW |
| DEX-related adverse effect | Number of studies  included | References of studies included | DEX  N | DEX (Mean or n/N) | Control  N | Control  (Mean or n/N) | Mean Difference  [95% Confidence Interval] | P-Value for statistical significance | P-Value for  heterogeneity | I^2^ Test for  heterogeneity | Quality of evidence  (GRADE) |
| Chronic pain | 1 | 7 | 33 | 1/33 | 32 | 8/32 | **-** | - | - | - | ⊕⊝⊝⊝, VERY LOW |
| Hypotension | 5 | 8,9,34,37,42 | 145 | 12/145 | 145 | 7/145 | 1.75 [0.67, 4.59] | 0.25 | 0.98 | 0% | ⊕⊕⊕⊝, MODERATE |
| Nausea and Vomiting | 11 | 7,8,9,34,36,37,38,40,41,42,43 | 298 | 49/298 | 297 | 59/297 | 0.79 [0.51, 1.23] | 0.29 | 0.94 | 0% | ⊕⊕⊕⊕, HIGH |
| Bradycardia | 5 | 8,9,34,37,42 | 145 | 13/145 | 145 | 10/145 | 1.37 [0.57, 3.31] | 0.49 | 0.90 | 0% | ⊕⊕⊕⊝, MODERATE |
